# Patient gender and rotator cuff surgery: are there differences in outcome?

**DOI:** 10.1186/s12891-021-04701-y

**Published:** 2021-09-30

**Authors:** Marlis T. Sabo, Justin LeBlanc, Kevin A. Hildebrand

**Affiliations:** 1 SCRUBS Research Unit, University of Calgary, 4448 Front St SE, AB T3M 1M4 Calgary, Canada; 2 Department of Surgery, University of Calgary, 1403 – 29th Street NW, Calgary, Canada

## Abstract

**Background:**

Although rotator cuff syndrome is common and extensively studied from the perspective of producing healed tendons, influence of gender on patient-reported outcomes is less well examined. As activity and role demands may vary widely between men and women, clarity on whether gender is an important factor in outcome would enhance patient education and expectation management. Our purpose was to determine if differences exist in patient-reported outcomes between men and women undergoing rotator cuff surgery.

**Methods:**

One hundred forty-eight participants (76 W:72 M) aged 35–75 undergoing surgery for unilateral symptomatic rotator cuff syndrome were followed for 12 months after surgery. Demographics, surgical data, and the Western Ontario Rotator Cuff (WORC) scores were collected. Surgery was performed by two fellowship-trained shoulder surgeons at a single site.

**Results:**

There were no gender-based differences in overall WORC score or subcategory scores by 12 months post-op. Pain scores were similar at all time points in men and women. Women were more likely to have dominant-arm surgery and had smaller rotator cuff tears than men. Complication rates were low, and satisfaction was high in both groups.

**Conclusion:**

Patient gender doesn’t appear to exert an important effect on patient-reported rotator cuff outcomes in this prospective cohort. Further work examining other covariates as well as the qualitative experience of going through rotator cuff repair should provide greater insight into factors that influence patient-reported outcomes.

**Supplementary Information:**

The online version contains supplementary material available at 10.1186/s12891-021-04701-y.

## Introduction

Approximately 180,000^1^ Canadian adults will develop symptomatic rotator cuff syndrome (a symptomatic rotator cuff tear, with or without accompanying long biceps tendon or acromioclavicular pathology) each year, experiencing the pain and disability that results [1]. Sufferers classically experience pain and weakness with reaching activities and fragmented sleep due to night pain [2]. The negative impact of rotator cuff syndrome on daily life varies, but some are unable to work, some are unable to be care-givers, and many experience loss of recreational activities important to their physical and emotional health.

The patient population facing symptomatic rotator cuff repairs is diverse: from roughly 30–80 years old, men and women, office workers and labourers, physically active and sedentary [3]. Much effort has focused on biological and technical factors to improve the rates of “successful” surgery. It is becoming increasingly clear, however, that addressing structural elements alone does not always produce the expected treatment outcomes from a patient perspective [4]. In addition to biological factors identified as important to structural outcomes such as increasing age, current smoking, and diabetes [5], patient-specific factors such as gender and psychosocial diversity may also have a role to play in patient-reported outcomes of rotator cuff surgery.

Despite gender being one of the most foundational demographic traits, it has rarely been explicitly researched as a primary factor in rotator cuff outcomes. It is important to appreciate that there is a distinction between a person’s gender and that person’s sex. The World Health Organization differentiates them as follows: “Gender refers to the characteristics of women, men, girls and boys that are socially constructed. This includes norms, behaviours and roles associated with being a woman, man, girl or boy, as well as relationships with each other.” Sex, then “refers to the different biological and physiological characteristics of females, males and intersex persons, such as chromosomes, hormones and reproductive organs.” [6].

Investigating potential influences of gender on rotator cuff outcomes is important because functioning of the upper extremities may impact gender-specific behaviors and activities of daily living differently for men and women. While more attention is being paid to this, the picture is incomplete. Previous studies on rotator cuff outcomes have not made a distinction between gender and sex, and some have not even provided any data regarding either factor. Others have conducted secondary analyses with patient “gender” examined for its role in outcomes [7–15] with little commentary on the significance of any related findings. This may be understandable because gender was not the primary focus of any of the foregoing studies. On the other hand, this does limit the ability to discuss the impact of patient gender in patient-reported outcomes of rotator cuff surgery.

However, a few studies have provided insight into gender and outcomes. Razmjou et al. [16] and Gibson et al. [17] both found *gender*-based differences in lifestyle scores on the Western Ontario Rotator Cuff Index in pre-operative rotator cuff patients. A prospective cohort from Daniels et al. [18] showed females had greater early VAS pain scores and poorer ASES scores until around 3 months post-operatively, and by 1 year post-op, outcomes in males and females were similar. Recent work by Pauly et al. [19] examined the relationship between patient sex, age, and rate of collagen-1 deposition, MRI healing at 1 year, and clinical scores at 1 year (ASES, WORC) and found no *sex*-based differences at 1 year. Continuing to flesh out the implications of gender-based differences in patient-reported outcomes may help construct future outcome studies and aid in more effective expectation management for patients.

Although the focus of this work will be on gender-based factors, physical factors such as patient height (which is typically different between males and females) could also influence the outcomes of a condition that affects overhead reaching activities.

The purpose of this study is to use a prospective cohort to explicitly examine the role of patient gender in patient-reported outcomes of rotator cuff surgery. We hypothesized that gender would not be a major influence on patient-reported outcomes. Potential confounding influence of height on outcomes was also examined.

## Methods

### Participants

**Fig. 1:**
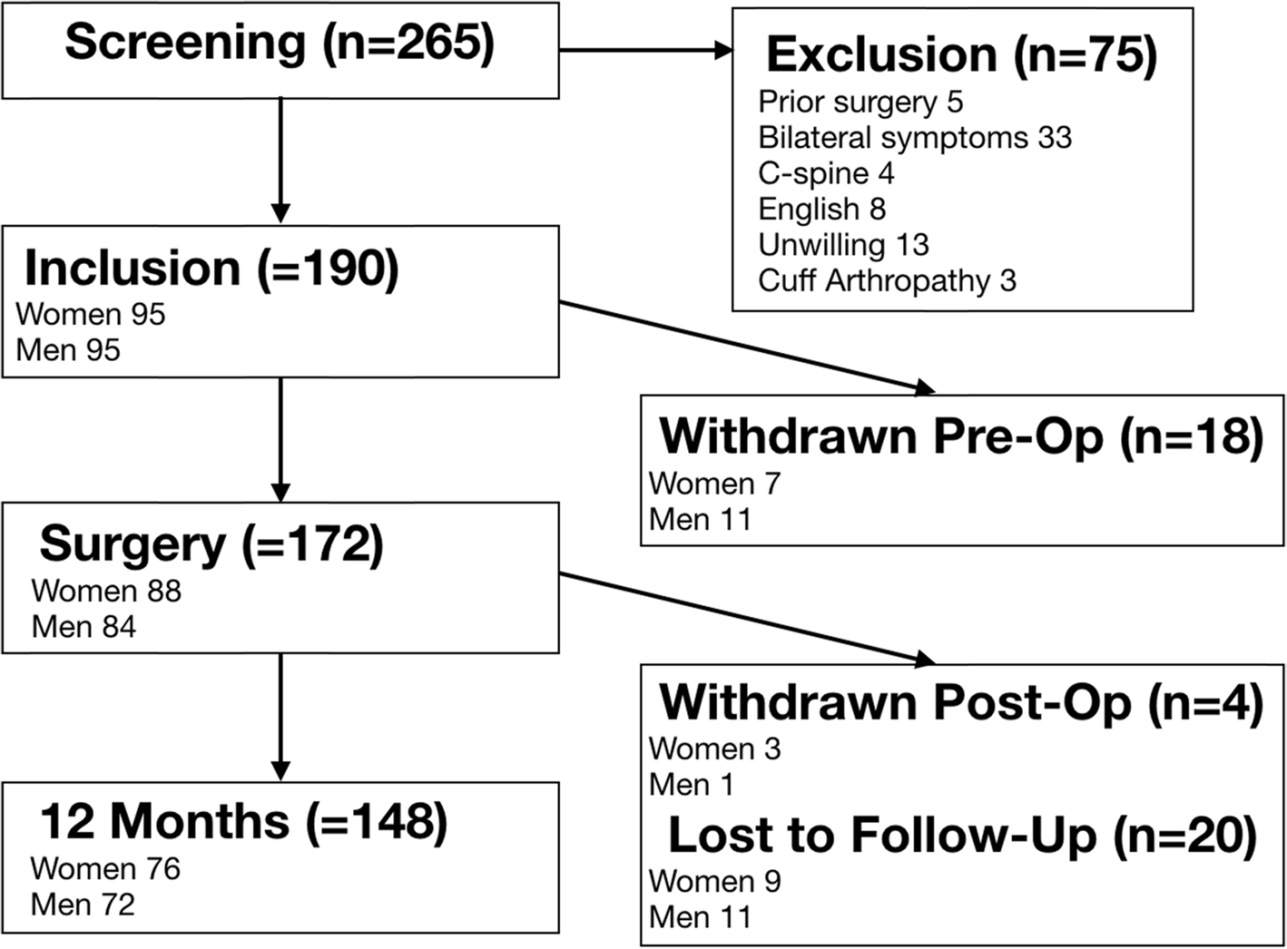
Flow diagram of patient recruitment and loss to follow-up

Eligible participants were aged 35–75 years undergoing elective surgery for unilateral partial and/or full-thickness rotator cuff tendon tears confirmed by ultrasound or MRI and were recruited by one of two fellowship-trained shoulder surgeons at a single-center between February 2016 and June 2017. Exclusion criteria were bilateral symptomatic rotator cuff disease, previous surgery on the operative shoulder, rotator cuff arthropathy, significant alternate sources of pain such as cervical spine disease or a chronic pain disorder (such as fibromyalgia or complex regional pain syndrome), and inability to complete questionnaires in English. Participants with known psychiatric diagnoses such as anxiety, depression, or related conditions were not excluded, nor were patients with potential gain issues, such as Workers’ Compensation board claims, litigation, or those injured in motor vehicle collisions. Transgendered participants were placed in the gender category that best matched their self-identified gender (affected 1 participant). No participants were excluded for being unable to identify with either of the two gender groups, although no openly non-binary or gender-fluid patients were screened during this time. Informed consent was obtained from all participants prior to enrollment. Figure 1 traces the path of the cohort from time of screening until final follow-up, accounting for where losses occurred. The project was reviewed and approved by the local Research Ethics Board (REB 15–1229) and was conducted according to the principles of the Declaration of Helsinki.

### Variables and outcome measures

Demographic data collected included gender, hand dominance, medical comorbidities, current medications, and smoking history. The disease-specific patient-reported outcome measure used was the Western Ontario Rotator Cuff score (WORC). The WORC score is a validated, self-reported measure of rotator cuff disease severity [20]. It consists of 21 questions in 5 domains, each with a 100 mm visual analogue scale (VAS). Higher total scores on the WORC indicate increased pain and functional disability. For the 2-week and 6-week visit, pain levels were determined by two 100 mm VAS scales drawn from the WORC-Pain domain to reduce patient questionnaire burden and to allow direct tracking of the evolution of pain over time. Post-operatively, VAS pain scores were collected at 2 and 6 weeks, while WORC scores were collected at 12 and 24 weeks, and 1 year post-operatively.

Rotator cuff tear characteristics were grouped based on intra-operative findings as follows: (1) any single partial-thickness tear in a single tendon, (2) partial-thickness tears in two or more tendons, (3) any full-thickness tear in a single tendon, (4) any full-thickness tear in a single tendon and any partial-thickness tear in a second tendon, and (5) full-thickness tears in two or more tendons.

Post-operative complications such as infection, nerve injury, excessive pain, excessive stiffness, failure of repair, and re-operation were also monitored for the duration of the study.

### Surgical intervention and post-operative care

Two fellowship-trained shoulder surgeons performed all surgical procedures. All patients presented for rotator cuff repair, with additional procedures relating to the long biceps tendon or acromioclavicular joint at the discretion of the surgeon based on clinical presentation.

All patients received pre-operative antibiotics and were treated most commonly with combined general and regional anesthesia, with a minority receiving either regional only or general anesthesia only. All procedures were conducted arthroscopically. Biceps tenodeses were conducted through arthroscopic or mini-open approaches depending on surgeon preference and clinical situation.

All patients received a diagnostic arthroscopy, a subacromial bursectomy and rotator cuff assessment. Two patients out of the cohort were found to have partial thickness tears smaller than anticipated based on pre-operative imaging and therefore received only an arthroscopic bursectomy.

Choice of anchors and type of repair were at the surgeon’s discretion and influenced by tear size, morphology, and mobility. Augmentation was used in 1 patient. Partial repairs were undertaken in situations where a full repair was not possible. Partial tears that were high grade were typically completed and repaired.

Post-operative care consisted of wearing a sling for 6 weeks, with active-assisted range of motion commenced at 2 weeks post-op. Two protocols were employed based on small-medium tears and large-massive tears. The progressions were similar, but the large-massive protocol progressed slightly more slowly than the small-medium program. These protocols are summarized in Additional file 1. Each patient selected a physiotherapist to guide rehab according to the protocol provided by the surgeon. Adjustments to progression through the protocol were also made at surgeon’s discretion based on patient progress and specific rehabilitation deficits. The large geographic area served by this center precluded having all patients see the same team of therapists.

Post-operative imaging of the operative shoulder was at the surgeon’s discretion based on clinical indication and was not conducted routinely as that does not reflect real-world clinical practice in the environment in which this study was conducted.

### Sample size and data analysis

Our primary endpoint was chosen to be the difference in WORC score at 12-month follow-up. Using the MCID and available psychometric data on the WORC, it was determined that we required 35 men and 35 women to complete 12-month follow-up to have 80% power to detect a difference of 11.5% (the MCID). Since additional subgroup analysis was desired, this sample was increased to allow detection of medium effect sizes in such analyses. For example, *n* = 134 would allow for 80% power to detect an f^2^ = 0.1 for a multiple linear regression analysis with 5 variables, while *n* = 138 would allow a 2-group 4-timepoint repeated measures ANOVA analysis to detect an effect size of 0.1 at 80% power. A practice audit revealed that a loss to follow-up of up to 30% would need to be planned for. With the aid of a dedicated research assistant, it was felt this could be reduced to about 25%. Thus, we aimed to recruit 92 men and 92 women for the cohort.

Descriptive statistical analysis was conducted with t-tests (for example, age, height, BMI) and chi-square tests (for example, occupation, comorbidities, tear pattern or size) as appropriate. Analysis of the patients not completing the study was performed with one-way ANOVA (age), Kruskal-Wallis H-test (WORC score), Fischer Exact tests (remainder of characteristics). Pearson product-moment correlation between height and WORC score was also performed. Repeated-measures ANOVA testing was used with gender and time as inputs. Several patients (20 of 148) had missing data points between time 0 and 12 months and so were excluded from repeated-measures analyses. Normality of the data distribution for the repeated measures ANOVA was confirmed through a histogram of the residuals demonstrating a normal distribution. Graph Pad (San Diego, CA) and R (version 3.6.3, 2020/02/29, R Foundation for Statistical Computing, Vienna, Austria) was used for the analyses. Statistical advice was sought from affiliated biostatisticians prior to the commencement of the study for sample size determination (including the decisions about scaling the sample size as described)^2^ and for the higher-level analyses at the conclusion of the study (including the repeated measures ANOVA and discussion of the normality of the residuals).^3^

## Results

### Demographics and characteristics of the women and men

A total of 148 patients completed 12 months of follow-up (Fig. 1). Table 1 shows the relevant demographics of this cohort. Of note, the women were more likely to carry a past diagnosis of a mood disorder. Women were much more likely to be presenting for surgery on their dominant arm compared to men. There were similar types of occupation in both groups, and similar rates of smoking and diabetes. No differences between those withdrawing or being lost to follow-up and those completing the study were noted. The cohort represented a mixture of acute, acute-on-chronic, and chronic tears. For those with a clear onset of symptoms or injury (102 participants), the median time from symptoms to surgery was 15.5 months (range 1–112 months). There were no differences between those who did not complete the 12 months of follow-up and those who did with respect to age (*p* = 0.57), baseline WORC score (*p* = 0.72), secondary gain concerns (*p* = 0.65), tear size (*p* = 0.35), tear pattern (*p* = 0.48), smoking status (*p* = 0.22), gender (*p* = 0.46), dominant arm operated (*p* = 1.0), diabetes mellitus (*p* = 0.32), presence of a mood disorder (*p* = 0.66), or occupation type (*p* = 0.78).

**Table 1:** Demographic information for women and men completing 1 year follow-up

Characteristic	Women (***N*** = 76)	Men (***N*** = 72)	
**Age**	55.6 ± 8.4 (36–74)	54.5 ± 8.2 (35–69)	*p* = 0.42 (95% CI −1.6 to 3.8)
**Height (CM)**	164.5 ± 7.3 (146–185.4)	177.5 ± 7.6 (159–196)	*p* < 0.0001^#^ (95% CI − 15.4 to − 10.6)
**BMI (KG/M**^**2**^**)**	28.6 ± 5.6 (16.3–44.5)	28.9 ± 4.1 (18.5–38.3)	*p* = 0.7 (95% CI − 1.9 to 1.3)
**Work type/Status**			
Active, full duties	10	15	*p* = 0.11
Active, modified duties	5	11	
Active, on medical leave	5	9	
Sedentary, full duties	28	25	
Sedentary, modified duties	4	2	
Sedentary, on medical leave**	1	0	
Unemployed	6	1	
Disabled for other reasons**	1	0	
Retired, active, no limits	2	4	
Retired, active, limited	10	4	
Retired, sedentary	3	1	
Other**	1	0	
**Smoking status**			
Never	51	49	*p* = 0.88
Former	19	16	
Current	6	7	
**Diabetes mellitus**	2	6	*p* = 0.12
**Secondary gain**			
Workders comp	2	4	*p* = 0.37
Accident litigation	8	0	*p* < 0.02*
**Hand dominance**			
Right	68	68	*p* = 0.27
Left	8	4	
% Dominant side operated	86%	58%	*p* = 0.0002*^#^

Statistically significant differences between genders was noted for height, number of personal injury litigations, and percentage of dominants arms operated. *Denotes statistical significance *p* < 0.05. **Omitted from statistical comparison due to insufficient numbers in category

Table 2 shows the intra-operative findings. All tear patterns were represented, with superior and anterosuperior being the most commonly found. Women tended to have smaller tears than men in this cohort. “Complete in 1 of Multiple” refers to a situation in which a large to massive posterosuperior tear only allowed for a portion of infraspinatus to be repaired rather than, for example, all of infraspinatus but none of supraspinatus. A higher rate of partial repair in men was observed.

**Table 2:** Operative findings and interventions

Characteristic	Women	Men	
**Tear pattern**			
Superior	35	22	
Anterior	4	2	*p* = 0.22
Anterosuperior	24	22	
Posterosuperior	6	10	
Massive (3 OR MORE)	6	11	
**Tear size**			
Partial in any 1 tendon	22	4	
Partial in any 2 tendons	3	5	*p* = 0.01*
Full in any 1 tendon	16	20	
Full in any 1, partial in second tendon	8	10	
Full in any 2 tendons	18	17	
Full in > 2 tendons^a^	8	14	
**Repair achieved**			
Complete in all tears	73	61	*p* = 0.03*
Complete in 1 of multiple	1	2	
Partial repair	1	8	
**Biceps management**			
No treatment required	20	31	*p* = 0.16
Tenotomy	6	3	
Tenodesis	43	38	
**Distal clavicle management**			
No treatment required	69	67	*p* = 0.62
Arthroscopic excision	7	5	

The distribution of tear patterns was similar between genders, while women tended to have smaller tear sizes. Men were more likely to receive only a partial repair. There were no differences in ancillary procedures. *Denotes statistical significance *P* < 0.05. ^a^ Or any three tendons with at least 1 full-thickness tear

### Patient-reported rotator cuff outcomes

**Fig. 2:**
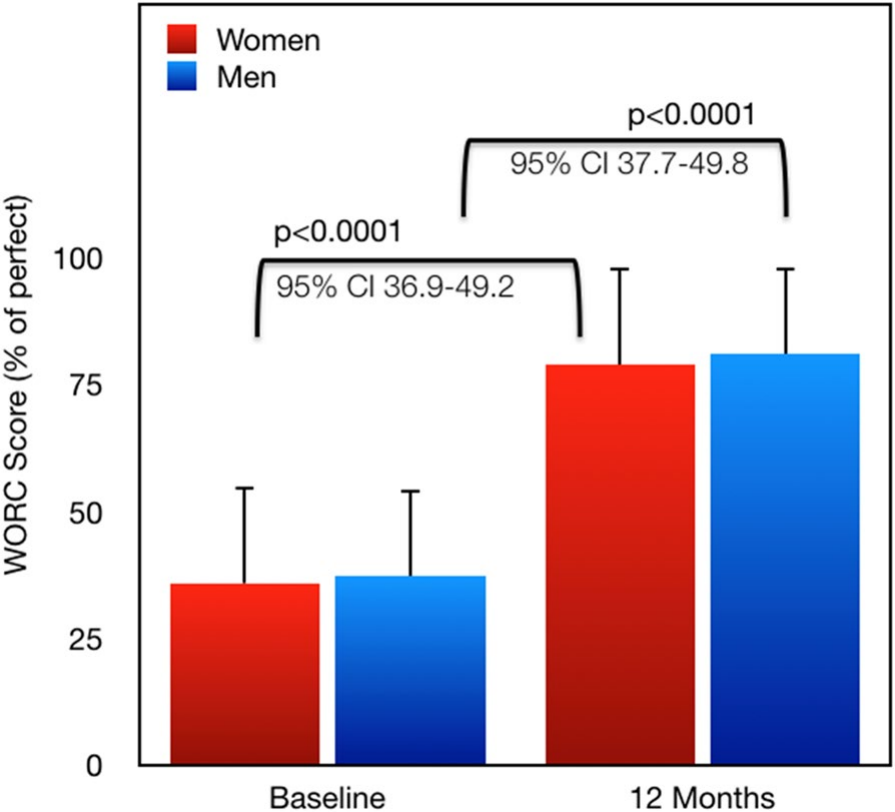
Western Ontario Rotator Cuff (WORC) Score before and after surgery in women and men. No differences were noted between genders at either time point. All values are shown mean ± standard deviation

**Fig. 3:**
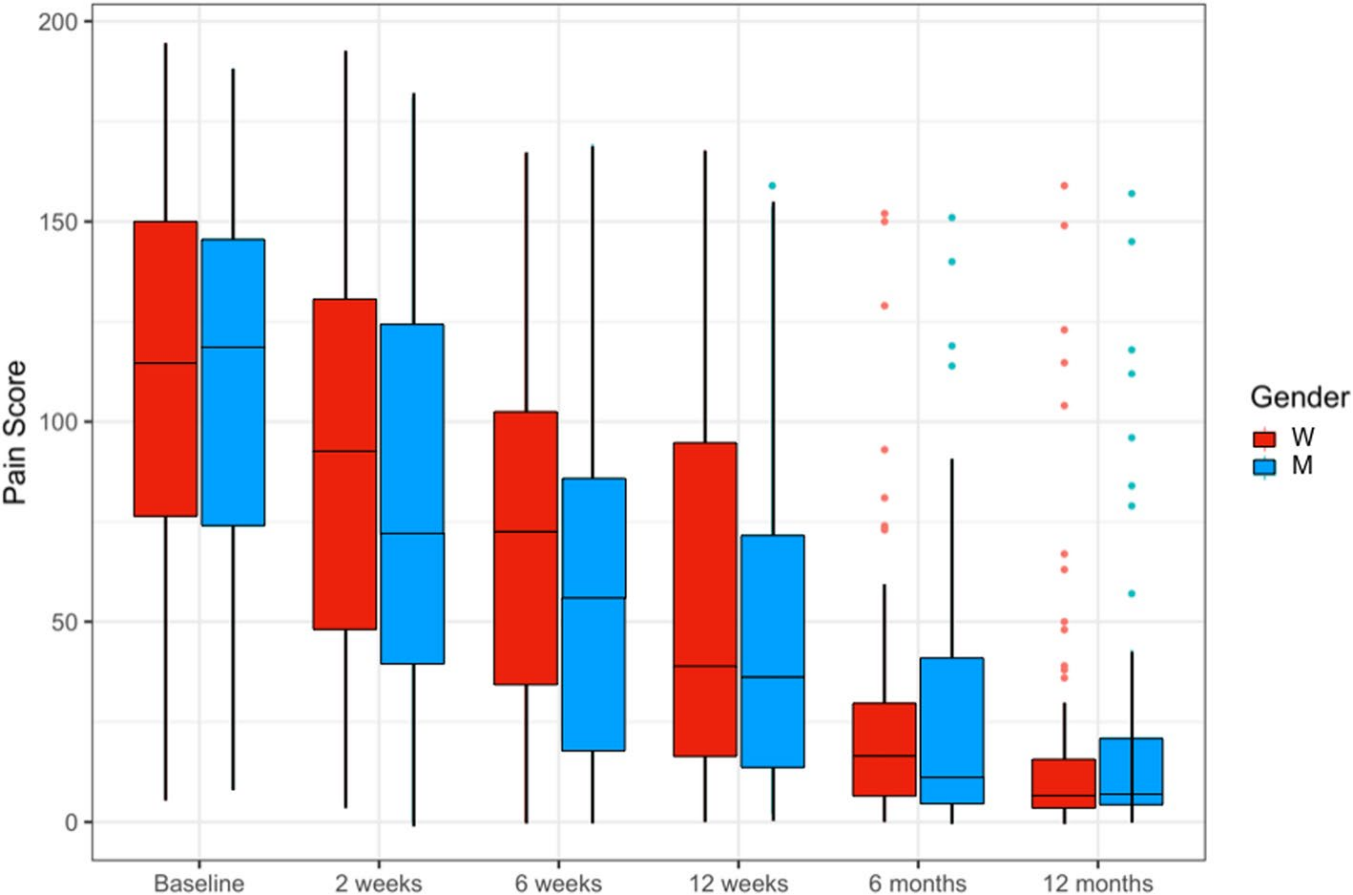
Pain scores in men and women over time after surgery. There were no differences between men and women at any time point as determined with a two-ways repeated measures ANOVA (*p* = 0.29, *n* = 126)

Fig. 2 shows the comparison of WORC score at baseline and at 12 months for men and women. Both groups improved substantially, but there were no differences between men and women at either time 0 or at 12 months follow-up. Figure 3 shows the progression of pain scores over time for both genders. This VAS was a summed total of reported sharp pain and dull pain taken from the WORC pain section. Again, both genders showed a substantial improvement in pain over the course of the post-operative year with no difference between groups at any time points.

The WORC score is divided into 5 domains that are assessed. No differences between men and women were noted for the Physical (*p* = 0.83, CI -7.29-5.83), Sports (*p* = 0.19, CI -2.90-14.21), Work (*p* = 0.7, CI -6.48-9.61), Lifestyle (*p* = 0.18, CI -2.17-11.37), or Emotions (*p* = 0.98, CI -7.42-7.18).

### Relationship between WORC score and tear pattern and size

The influence of intra-operative tear size on WORC score was also examined. Tear shape (anterior, anterosuperior, superior, posterosuperior, and massive) was not associated with significant differences in WORC score at baseline (*p* = 0.84) or at 12 months post-operative (*p* = 0.48). Tear size was similarly not associated with significant differences at baseline (*p* = 0.98) or 12 months post-operatively (*p* = 0.82).

### Relationship between patient height and WORC score

An examination of the relationship between height and WORC score revealed that although women were significantly shorter than men in this cohort (Table 1), patient height did not prove to have an important influence on WORC score (R = 0.035).

### Gender, satisfaction, and patient feedback

At the 12-month follow-up, patients were asked three yes/no questions about their experience. First, whether they were satisfied with the results of their rotator cuff surgery (W:93% yes, M:91% yes). Second, if they knew at time of electing for surgery what they know now, would they have the surgery again(W:92% yes, M:98% yes)? Third, if a friend or family member had a similar shoulder problem, would they recommend surgery to them (W:97% yes, M:100% yes)? No significant differences were noted between men and women for each of the three questions, although there was a trend towards women being less likely to want to go through surgery again (*p* = 0.07).

### Complications

Complication rate was low for the cohort. There were no deep infections. Two patients experienced neurological symptoms in the lower arm, one of which had partially improved at 1 year. One underwent revision surgery for a failed biceps tenodesis. Two underwent reoperation with a non-study surgeon to receive a Superior Capsular Reconstruction. In addition, a further 5 patients had a documented repair failure, most of whom underwent revision surgery. One retear had a documented fall before 12 weeks post-op. Three patients received corticosteroid injection therapy during the year after surgery.

## Discussion

This study has demonstrated no significant difference in patient-reported outcome of rotator cuff surgery between men and women 1 year after surgery. It has also demonstrated no significant relationship between tear pattern or tear size and patient-reported outcome at 1 year following surgery. Women were more likely to be undergoing surgery on their dominant arm, more likely to have a smaller tear size, and more likely to have had a full rotator cuff repair than men. There were more women with litigation related to their shoulder problem than men. Both women and men experienced statistically and clinically significant improvement in WORC score from pre-op to final follow-up.

### Patient height and WORC score

Because people of a wide range of height have to negotiate standardized environments at home and in the workplace, we did examine the relationship between patient height and WORC score. Height is not evenly distributed across genders. Our hypothesis was that since persons of smaller stature likely spend more of their activity with their arms at/above shoulder height, their reported outcomes may reflect greater individual activity limitations related to rotator cuff disease even after surgery. This did not prove to be the case here.

### Gender and outcome

Existing literature examining the relationship between gender and outcome has mainly dealt with gender as a subanalysis, with mixed findings. Table 3 provides a summary of the relevant literature commenting on the outcome of rotator cuff surgery in female patients derived from secondary analyses. Despite the liberal use of the term “gender” in these studies, the author intent was more consistent with “sex” in all but the Ramzjou study [16]. Comparison between studies is challenging because of the diversity of outcome measures used, but the trend was towards poorer outcomes in female patients.

**Table 3:** Summary of Outcomes of Rotator Cuff Surgery

Author/Year	Sex or Gender?	N	Purpose	Instruments	Findings
Gartsman/1998 [9]	Sex/Female	24F:26M	Outcome of arthroscopic cuff repairs, correlating SF-36 scores with shoulder scores	Constant, ASES, UCLA, SF-36	Females had greater improvements in scores (ASES, Constant) from pre- to post-op.
Romeo/1999 [6]	Sex/Female	28F:48M	Outcomes of open cuff surgery at mean 4.5 years post-op	Constant, UCLA, SST	Age and sex intersected so older females did worse on Constant, but not clinically significant.
Cofield/2001 [10]	Sex/Female	33F:72M	Outcome of open rotator cuff repairs	Neer system, range of motion, strength, satisfaction	Females had worse pain scores, range of motion, and result ratings
Watson & Sonnabend/ 2002 [12]	Sex/Female	262F:405M	Outcomes of full-thickness cuff repairs	Adapted SST	Females more likely to report improvement, especially with work.
Harryman/2003 [8]	Sex/Female	137F:196M	Examination of extra-shoulder factors on outcome (ie: age, gender, comorbidities)	Simple Shoulder Test (SST)	In this pre-op study, female sex was correlated to poorer SST scores than male sex.
Milano/2010 [7]	Sex/Female	35F:66M	Comparison of rotator cuff repair using metal vs bioabsorbable anchors	DASH, Work-DASH, Constant	Sex was not associated with post-operative outcomes
Grasso/2009 [14]	Sex/Female	38F:34M	RCT of single vs double row repairs at 2 years	DASH, Work-DASH, Constant	Female sex associated with lower DASH and strength scores at 2 years.
Oh/2012 [13]	Sex/Female	57F:61M	Explore relationship between pre-op concern and expectation of post-op recovery on post-op outcome	MODEMS score, SST, Constant	Female sex was associated with the “high concern” group; they “high concern” group had poorer outcomes (ie: less improvement from pre-op state).
Chung/2012 [11]	Sex/Female	158F:151M	Measure HRQOL^a^ minimum 1 year after arthroscopic cuff repair	SF-36, SST, Constant, ASES	Female sex associated with lower SF-36 physical and mental component scores. No sex-based data on shoulder-specific scores.
Razmjou/2006 [15]	Gender/ Woman^b^	108F:171M	Effect of age and gender on pre-operative scores	WORC Score	Females had poorer scores on Emotions subscale, with trend to difference on Lifestyle scale also
Razmjou/2011 [20]	Gender/ Woman	85 W:85 M	Gender-based effects on 6-month outcomes of rotator cuff related surgery	WORC Score, ASES, QuickDASH	Women had poorer pre- and post-operative shoulder outcome scores.
Daniels/2019 [17]	Sex/Woman^c^	130F:153M	Effect of sex on post-operative outcomes and narcotic use	VAS pain, ASES, narcotic use at 2-weeks post-op	Women had higher initial pain scores, and higher 2-week narcotic use. Final outcomes similar.

^a^Health Related Quality of Life (HRQOL)

^b^Sex and gender terminology was used interchangeably in this paper making it challenging to discern the intended category choices. Despite this, the usage is most consistent with “sex” rather than “gender”

^c^Similar to the above, mixed terminology makes it difficult to discern the authors’ intent

One closely relevant work on gender and rotator cuff disease to this current study is from Ramzjou et al. in 2006 [16]. They assessed 279 patients (108 women) undergoing rotator cuff surgery. They noted differences in prevalence of emotional disturbance in women (using the WORC-Emotions scale), and the structural pathology was distributed differently between younger men and women. This difference faded with age. They also noted a trend towards differences in the WORC-Lifestyle scale by gender. This difference was also noted by Gibson et al. in a more recent investigation [17]. The Ramzjou paper proposed that there may be gender-based role activities that account for this difference (such as different grooming and dressing practices) [16].

In a subsequent report from Ramzjou et al., women were found to have poorer pre and post-op WORC scores than the men in the cohort [21]. One difference between the cohorts was that patients undergoing decompression-only as the intended procedure were included (whereas our cohort was intended to include a rotator cuff repair from enrollment). This may be important as decompression surgery is not clinically significantly better than non-operative treatment [22], thereby potentially performing differently from a rotator cuff repair. More women in the 170-patient cohort had decompression-only. Their cohort had similar tear sizes between genders, where women in this cohort tended to have tears confined to the supraspinatus. Their post-operative outcomes were collected at 6 months after surgery, whereas this cohort was followed for 1 year. In contrast to the Ramzjou cohort, this cohort did not note gender-based differences in the WORC total score or subscores at the 1-year follow-up. It is not possible to directly compare gender-based satisfaction from this cohort to the Razmjou cohort [21] as they approached this question differently. Further work will delve into potential explanations for why this potential difference might be so.

A second prospective cohort followed by Daniels et al. [18] included 283 patients (130F:153M) for 1 year using the ASES score as their primary disease-specific outcome measure. They found differences at baseline and for the first 3 months after rotator cuff repair between males and females, which disappeared by 1 year. Our cohort affirms their findings of similar 1-year results in both genders. It is possible that their larger sample size allowed for a smaller effect size to be statistically significant. In our cohort, small differences in VAS pain scores could be seen, but these were neither statistically nor clinically significant. Literature on patient sex and sensitivity to various clinical and experimental pain modalities show mixed results, but tend to favor females being more sensitive to painful stimuli, making this variation plausible [23].

In view of this, some questions arise. Do women have more symptoms for a given tear size than men, or could it be that tear size isn’t well correlated to patient-reported symptoms? We looked at tear size by number of tendons involved, and by tear pattern, and there were no differences in WORC score in either analysis. This is consistent with the work by Wylie et al. which suggested that tear size and symptoms may not cohere well [24]. Interestingly, although their work did show relation between tear size and VAS function scales, this current study did not relate tendon involvement to WORC score. Differing techniques of measurement and different instruments limit direct comparisons in this domain. This follow-up question is well-suited to a case-control design rather than a cohort design.

We also noted a striking difference between dominant operative arms between the men and women. Women were much more likely to be undergoing surgery on their dominant arm (86% vs 58%). Ramzjou et al. [21] didn’t explicitly state the percentage of dominant arms operated by gender, but more women were right-handed and underwent right-sided surgery. Again, direct comparison is challenging due to different ways of presenting the data. This again may gesture towards gender-based differences in activity demands.

Although Gibson et al. noted a small but significant difference in WORC-Lifestyle scores by gender in the pre-operative analysis of this cohort [17], this difference is no longer statistically or clinically significant at 1 year follow-up. It would be interesting to determine whether this represents a consistent return of function to baseline or pre-morbid activity, or whether this represents a long-term acceptance and adaptation of lifestyle activities as part of a holistic post-operative recovery.

Whether meaningful differences in outcomes of rotator cuff surgery depends on patient gender is likely subject to one of two realities. First, as this current study and others have shown, no significant differences at minimum 1-year follow-up exists and this is a true finding. Alternatively, our clinical tools are not well configured to detect gender-based differences in patient-reported outcomes. Furtado et al. reflect on the short-WORC and the variably applicability of items such as dressing/undressing and hair styling across genders [25]. Whether enough gender difference exists to merit rethinking our patient-reported outcomes is beyond the scope of this study, and assuming no important differences exist may be premature.

### Sex, gender, and the future of outcomes research

As our understanding of sex and gender becomes more developed and substantially more complex, it is worth considering how outcomes research should approach this important issue. The Canadian Institutes for Health Research [26] actively encourages consideration of sex and gender in research design. Outcome measures to assess gender now exist.

The intent of this current work was explicitly to approach outcomes based on gender, not sex. An argument can be made to approach patient-reported outcomes on a gender basis given that they quantify lived experience moreso than a physiologic or structural outcome. However, this is a blunt distinction that doesn’t provide much guidance on how to clearly and consistently decide between gender and sex, nor does it help with adequate inclusion of patients and participants who do not identify as man or woman. It is hoped that with time, some clarity may be added to this complex discussion. In the meantime, a step forward is to be conscious that sex and gender are not interchangeable terms and this should be deliberately addressed in study design.

### Limitations

This study was appropriately powered for its primary endpoint, but may be underpowered for the subanalyses. There are several limitations. A pragmatic approach was taken to have this study reflect clinical practice as much as possible. It is not feasible or practical to continue follow-up for patients past 1 year. There is emerging evidence to suggest that longer follow-up may not be fruitful^15^. Longer follow-up might reveal improved WORC scores beyond those reported, but whether those differences are clinically meaningful to offset the potential exposure of more repair failures or retears is unclear.

Second, it is not feasible to obtain timely pre-operative MRI scans on all patients prior to rotator cuff surgery in the setting of this study. It is also not feasible to re-image all post-operative patients with MRI. While post-operative imaging was at surgeon discretion based on clinical indications, the final status of the repairs at 12 months has not been confirmed across all participants. Repair status was not a primary outcome of this study, but it does limit ability to comment on asymptomatic or mildly symptomatic retears.

Third, we elected to use categories rather than measurements of tear size. Because of the mixed imaging modalities pre-operatively, tendon involvement was obtained from the operative record. While this reduces measurement errors between assessors and avoids classification errors based on highly variable patient and tendon footprint size, it does complicate ability to compare directly with studies where tear length/area is reported in detail. Amongst existing classifications of rotator cuff tear size, some focus more on number/location of involved tendons more than absolute linear size. For example, Wylie et al.^24^ did use both quantitative and qualitative data for tear size.

Fourth, this was a single-center study. While this increases the consistency of surgical interventions, it could limit generalizability.

Fifth, there was the potential for heterogeneity in post-operative rehabilitation amongst the cohort. Despite all patients being provided with the same protocols, we were not able to monitor all the physiotherapists that could have had contact during the time of the study. It is possible that this introduces a factor not quantified by the study.

Finally, this cohort contains a high proportion of sedentary workers which may limit the generalizability of these results to heavy laborers or athletes of either gender.

## Conclusions

In this prospective cohort of 148 patients, we have shown that patient gender does not have an important effect on WORC scores 1 year after arthroscopic rotator cuff repair. In comparing the men and women in this study, we do note that women were more likely to be having surgery on a dominant arm and tended to have smaller structural pathology than the participating men. Further work examining other covariates as well as the qualitative experience of going through rotator cuff repair should provide greater insight into factors that influence patient-reported outcomes.

## Supplementary Information

**Additional file 1.**

## Data Availability

All data generated and analysed for this report are available as deidentified data upon reasonable request from the corresponding author.

## Footnotes

1 Calculated from 2016 Stats Can Data http://www.statcan.gc.ca/tables-tableaux/sum-som/l01/cst01/demo10a-eng.htm

2 The team would like to thank Dr. E Oddonne-Paolucci for her advice on sample size determination.

3 The team would like to thank Dr. K Rondeau for her assistance with final analysis.
