# Supplementary material for: Patient gender and rotator cuff surgery: are there differences in outcome?
Source: BMC Musculoskelet Disord. 2021 Sep 30;22:838. doi: 10.1186/s12891-021-04701-y (PMC8485499; doi:10.1186/s12891-021-04701-y)
Supplement: Supplementary file 1 — Additional file 1. [file 12891_2021_4701_MOESM1_ESM.docx]

## Appendix 1: Physiotherapy Protocols

**table 1.1: Comparison of physiotherapy protocols**

| **Time Post-Surgery** | **Small-to-Medium Tear** | **Large-to-Massive Tear** |
| --- | --- | --- |
| **0-2 weeks** | Ice, gentle massage around shoulder, elbow*/wrist/hand movement, scapular setting/posture. | |
| **2-4 weeks** | Pendular exercises, assisted motion in supine for internal and external rotation, forward elevation to 90-120º with flexed elbow | |
| **4-6 weeks** | Continued assisted motion with stick in supine, proprioception exercises below 70º forward elevation | |
| **6-10 weeks** | Transition to active motion with focus on scapular control, proprioception exercises in higher elevation angles, advanced stretching (ie: Child’s pose, table glides) | |
| **10-12 weeks** | Initiation of resistance exercise, initially below shoulder height, small weights and bands |  |
| **12 weeks onward** | Progression of resistance exercise into abducted or shoulder-height positions. Sport/activity-specific training, progression to gym setting, etc. | Initiation of resistance exercise, initially below shoulder height, small weights and bands.  Progression of resistance exercise into abducted or shoulder-height positions. Sport/activity-specific training, progression to gym setting, etc. |
| **Approximately 6 months** | Progress to independent work and reintroduction of all activities as able. | |

Allocation of a patient to one protocol or other was based on tear size (> 3 cm typically large-massive program) and surgeon impression of tissue quality or bone fixation strength. Both protocols arrive at a similar place by 6 months post-op.
